# Synthetic Lethality by Co-Inhibition of Androgen Receptor and Polyadenosine Diphosphate-Ribose in Metastatic Prostate Cancer

**DOI:** 10.3390/ijms25010078

**Published:** 2023-12-20

**Authors:** Mariangela Calabrese, Isabella Saporita, Fabio Turco, Silke Gillessen, Elena Castro, Ursula Maria Vogl, Rosario Francesco Di Stefano, Federica Maria Carfì, Stefano Poletto, Giovanni Farinea, Marcello Tucci, Consuelo Buttigliero

**Affiliations:** 1 Department of Oncology, University of Turin, AOU San Luigi Gonzaga, 10043 Orbassano, Italy; mariangela.calabrese@unito.it (M.C.); isabella.saporita@unito.it (I.S.); turcofabio9@gmail.com (F.T.); rosario-distefano@virgilio.it (R.F.D.S.); federicamaria.carfi@unito.it (F.M.C.); stefano.poletto@unito.it (S.P.); giovanni.farinea@unito.it (G.F.); 2 Ente Ospedaliero Cantonale—Istituto Oncologico della Svizzera Italiana, 6500 Bellinzona, Switzerland; silke.gillessensommer@eoc.ch (S.G.); ursula.vogl@eoc.ch (U.M.V.); 3 Department of Medical Oncology, Faculty of Biomedical Sciences, Università della Svizzera Italiana, 6900 Lugano, Switzerland; 4 Hospital Universitario 12 de Octubre, 28041 Madrid, Spain; elena.castro@ibima.eu; 5 Department of Medical Oncology, Cardinal Massaia Hospital, 14100 Asti, Italy; marcello.tucci@gmail.com

**Keywords:** prostate cancer, androgen receptor pathway inhibitor, PARP inhibitors, synthetic lethality, androgen receptor, homologous recombination repair, BRCA mutation

## Abstract

Androgen receptor pathway inhibitors (ARPI) and polyadenosine diphosphate-ribose inhibitors (PARPi) are part of the standard of care in patients with metastatic castration-resistant prostate cancer (mCRPC). There is biological evidence that the association of ARPI and PARPi could have a synergistic effect; therefore, several ongoing clinical trials are investigating the efficacy of this combination with preliminary results that are not perfectly concordant in identifying patients who can obtain the most benefit from this therapeutic option. The purpose of this review is to describe the PARPi mechanisms of action and to analyze the biological mechanisms behind the interplay between the androgen receptor and the PARPi system to better understand the rationale of the ARPI + PARPi combinations. Furthermore, we will summarize the preliminary results of the ongoing studies on these combinations, trying to understand in which patients to apply. Finally, we will discuss the clinical implications of this combination and its possible future perspectives.

## 1. Introduction

Prostate cancer (PC) is the most common tumor in men worldwide and the second cause of cancer-related death after lung cancer [1]. While localized PC is associated with more favorable outcomes, metastatic PC is considered incurable, with a 5-year survival rate of 32% according to the American Cancer Society (data based on patients diagnosed with PC between 2012 and 2018) [2].

PC arises as a hormone-driven tumor, since its growth is strongly induced by androgens; consequently, androgen deprivation therapy (ADT) has been the standard of care in patients with metastatic hormone-sensitive PC (mHSPC) for decades [3].

Since 2015, the results of several trials showed impressive survival advantages for the intensification of systemic treatment in the mHSPC setting with the addition of either chemotherapy [4,5,6] or androgen receptor pathway inhibitors (ARPI) to ADT [7,8,9,10,11]. A further revolution in this setting occurred when the results of triplet therapy were presented: recently, the phase 3 trials PEACE-1 and ARASENS showed an overall survival (OS) benefit combining ADT, docetaxel and ARPI (abiraterone or darolutamide) compared to ADT and docetaxel in patients with mHSPC [12,13]. Following the results of these studies, combination therapy represents the standard of care in mHSPC patients.

Unfortunately, despite these therapeutic advances, disease will progress and become resistant to castration (metastatic castration-resistant PC, mCRPC) [14].

Despite the onset of castration resistance, it is well known that PC cells do not become completely hormone-independent, and re-activation of androgen receptor (AR) signaling may be one of the key drivers of disease progression in the mCRPC setting [3]. For this reason, patients with mCRPC continue ADT for their entire life, and a stronger inhibition of the AR pathway through the addition of abiraterone or enzalutamide to ADT was shown to improve outcomes both in the pre- [15,16] and post-docetaxel setting [17,18]. Docetaxel represents another possible therapeutic option in patients with mCRPC, since it has been demonstrated to be superior to the previous standard of care treatment at that time, represented by mitoxantrone [19,20]. Cabazitaxel has also shown to be effective in the mCRPC setting and particularly in patients previously treated with docetaxel [21]. Radiopharmaceutical agents have also been demonstrated to improve survival in selected mCRPC patients. In particular, radium-223 was shown to improve OS in patients with symptomatic bone metastases and without visceral metastasis [22], while ^177^-lutetium-PSMA-617 was shown to be effective in patients with a prostate-specific membrane antigen (PSMA)—positron emission tomography (PET) (PSMA–PET) positive disease who failed a previous treatment with docetaxel and ARPI [23,24].

In recent years, molecularly targeted approaches have also been developed in PC. Deleterious alterations in genes involved in homologous recombination repair (HRR) are usually associated with an aggressive phenotype of PC but might also convey sensitivity to treatment with polyadenosine diphosphate-ribose (PARP) inhibitors (PARPi) through a mechanism of “synthetic lethality”, a phenomenon which occurs when the combination of two genetic events results in cell death, whereas a deficiency of only one of these genes does not [25]. PARPi are molecules targeting a specific DNA damage repair system, leading tumoral cells to irreparable injury and subsequent death [26,27,28]. In PC, olaparib is approved by the European Medicines Agency (EMA) in patients with mCRPC and BRCA 1/2 mutations (germline and/or somatic) who have progressed following prior therapy, including ARPI [29]. The approval of olaparib was based on the results of the phase 3 PROFOUND trial in which olaparib was shown to improve OS in patients with mCRPC and HRR gene alterations, especially in those with a BRCA 1/2 mutation [30]. Another PARPi approved by the US Food and Drug Administration (FDA) but not by the EMA is rucaparib. Its approval is limited to mCRPC patients with BRCA1 or BRCA2 mutations who have progressed after at least one ARPI and one taxane-based chemotherapy, following the results of a phase II, single-arm study (TRITON 2) [31].

PARP inhibition has been reported to increase activity of ARPI via AR-dependent transcription, and ARPI induces HRR deficiency, increasing the susceptibility to PARPi, thus providing the idea of a synthetic lethality interaction and a strong pre-clinical rationale to test this combination both in patients with mutations in the HRR genes and in patients without them [32].

In this review, we will discuss the biological rationale and available clinical data of the ARPI and PARPi combination treatments and will try to understand if this combination could represent a possible therapeutic option in the future for the treatment of patients with metastatic PC (mPC).

## 2. Homologous Recombination Repair Pathway in Prostate Cancer

DNA damage is a well-known critical factor in cancer development and progression [33]. DNA damage response (DDR) and repair pathways perform the essential role to maintain genomic integrity and stability. Inefficient DNA repair is a driving force behind cancer establishment and evolution [34]. DDR pathways are generally classified into single-stranded DNA (ssDNA) repair and double-stranded DNA (dsDNA) repair pathways based on their mechanism of action. The DNA repair process regulates several steps, including the DNA lesion detection, the DNA repair factors recruitment around the damaged region, and repair through DNA biosynthesis [35,36]. The different DDR pathways are mutually interconnected, although different pathways are specialized for repairing specific types of DNA damage: single-strand breaks (SSBs) are typically repaired by base excision repair pathways such as PARP1, DNA mismatches generated during DNA replication are primarily corrected through the mismatch repair (MMR) pathway, and double-strand breaks (DSBs) are repaired by the nonhomologous end joining (NHEJ) system and the homologous recombination repair (HRR) pathway [37,38,39].

Alterations in DDR genes have been reported in 10% of localized prostate tumors and in almost a third of metastatic cases [40,41]. HRR is the DDR pathway most frequently altered in prostate cancer. In 28% of the samples analyzed in the PROFound study, at least one HRR alteration was found. The most frequently altered gene was BRCA2 (8.7%), followed by CDK12 (6.3%), ATM (5.9%), CHEK2 (1.2%), and BRCA1 (1%). Co-occurring aberrations in two or more HRR genes were identified in 2.2% of cases [30]. Analyses of paired samples from primary tumors and metastases at the time of castration resistance has shown no difference in the prevalence of DDR alterations, suggesting that these events occur early in tumorigenesis [42,43,44].

A significant proportion of HRR alterations found in prostate tumors have a germline origin [45,46], including 50% of the mutations in BRCA2. On the other hand, up to 12% of the patients who develop metastatic prostate cancer harbor germline mutations in cancer-predisposition genes [47,48]. Despite differences in the absolute frequency reported related to the different genetic background, BRCA2 is the gene most frequently affected by germline mutations across studies populations, followed by ATM. Germline BRCA2 mutations are a well-stablished poor prognosis factor for localized and advanced prostate cancer [49,50,51]. The impact of germline alterations in other HRR genes is less well stablished. A recent study presented at ASCO 2023 suggests that somatic BRCA2 alterations would have a detrimental impact on prognosis similar to those off germline origin [52]. The endpoint of the first cohort of the Capture study was to evaluate the association between presence of somatic/germline HRR gene alterations and outcomes in mCRPC patients receiving first line treatment, stratified by BRCA mutational status. In this large analysis, patients with mutations in BRCA1/2 had worse radiological progression-free survival (rPFS) and OS compared to patients without HRR mutations (HR for rPFS: 1.70, 95% CI: 1.32–2.19, *p* < 0.001; HR for OS: 1.95, 95% CI: 1.55–2.45, *p* < 0.001, respectively) and to patients with HRR non-BRCA mutations (HR for rPFS: 1.34, 95% CI: 0.98–1.81, *p* = 0.06; HR for OS: 1.40, 95% CI: 1.06–1.84, *p* = 0.017) [52].

In addition, HRR mutations have a therapeutic implication as they may sensitize patients to platinum-based chemotherapy [53,54] and target therapies, such as PARPi. Furthermore, it would appear that patients with the mCRPC and BRCA2 mutation are more sensitive to treatment with ARPI than to taxanes, even if more evidence is needed to support this hypothesis [48].

## 3. Mechanism of Action of PARP Inhibitors

**Figure 1:**
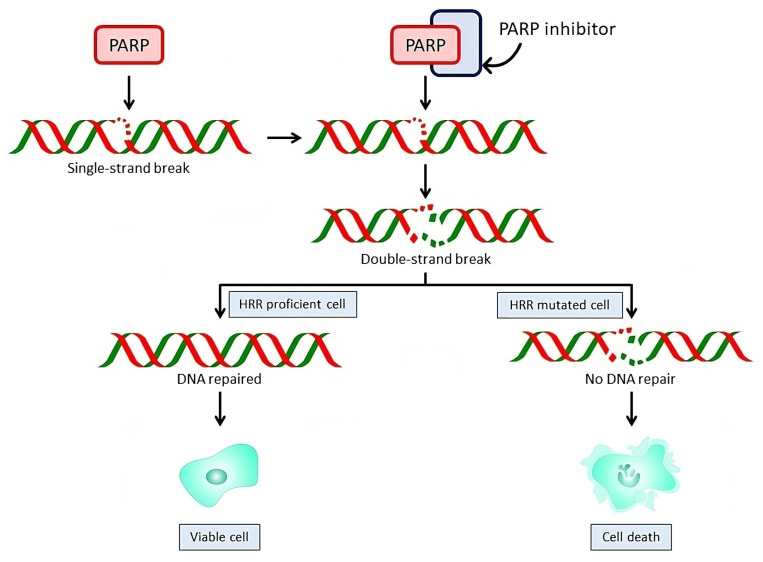
Mechanism of action of PARPi. PARP inhibition prevents single-strand breaks (SSBs) from repairing, leading to double strand breaks (DSBs) that will be repaired by homologous recombination repair (HRR) pathways. While in HRR proficient cells this DNA repair mechanism is functional and keeps the cell viable, in HRR-deficient cells, DNA cannot be repaired, resulting in cell death.

The PARP family of enzymes plays an essential role in DNA damage response, participating in early stages of SSBs repair. The PARP family includes 17 members that can be classified into different groups depending on their motifs and functions. Among them, PARP1 is the most abundant and was the first and most extensively studied enzyme recognized to play a crucial role in SSBs repair [55] (Figure 1). SSBs occur continuously as a consequence of oxidative stress, radiotherapy, UV light, alkylation products, or other external sources of damage. An SSB leads to PARP recruitment and activation. PARP1 and PARP2 cleave nicotinamide adenine dinucleotide and attach multiple ADP-ribose units to target proteins, including themselves. The consequence is a highly negatively charged protein, which results in the unwinding of the DNA strands and recruitment of proteins on the damaged DNA through the base-excision repair process [56]. When SSBs occur in the presence of a PARP inhibitor, PARP1 binds to DNA damage sites and remains trapped on the chromatin; SSBs cannot be repaired, resulting in the formation of DNA single-strand gaps that can be encountered by replication forks. When that happens, replication fork arrests and SSBs degenerate into DSBs. Normally, DSBs can be repaired by the HRR pathway, leading to replication fork restart and cell survival. In the absence of functional HRR pathway components, due to somatic or germline mutations, the replication fork cannot be restarted, causing persistent chromatid breaks. These breaks are repaired by alternative error-prone DSB repair mechanisms (NHEJ), causing large numbers of chromatid aberrations and pushing the cell towards death [26].

This means that PARP inhibition does not cause cell lethality by itself, as the cell has an intact HRR pathway for DNA repair, but it works through “synthetic lethality”, a phenomenon where loss of either of the two genes is not lethal per se, but concomitant inactivation leads to cell death [57].

Therefore, given that BRCA1/2 mutated tumor cells have disrupted HRR activity, the replication forks are unable to be repaired, and cell death occurs. This explains why cancer cells deficient in BRCA1 and BRCA2 are hypersensitive to PARPi.

PARPi are a novel class of anti-cancer therapies that compete with NAD+ for the catalytically active site of PARP molecules. In addition, PARPi are able to trap PARP1 at the level of the SSB and thus prevent the repair [58]. PARPi in clinical use have a different ability to trap PARP1, for example the most potent is talazoparib, with the ability to trap PARP1 100 times more efficiently than niraparib [58,59,60].

PARP’s functions are numerous and involve transcription, apoptosis regulation, and immunity modulation other than DNA repair. Thus, the antitumor action of PARPi could also be associated with these functions [55,61,62].

Since PARP has a key role in repairing DNA damage, PARPi were first developed as sensitizers of DNA damaging therapies such as ionizing radiation therapy and chemotherapy [63]. Only afterwards was PARPi’s therapeutic potential demonstrated in BRCA-deficient cells, which being defective in HRR system are highly sensitive to PARP inhibition in a synthetically lethal interaction [27]. Initial reports described that BRCA1−/− or BRCA2−/− cell lines displayed a 60- to 1000-fold greater sensitivity to olaparib and talazoparib precursors than BRCA+/+ cell lines. These observations have been replicated numerous times with other PARPi [60].

## 4. PARP Inhibitor Monotherapy in Metastatic Castration-Resistant Prostate Cancer

The first PARPi approved by the FDA in mCRPC harboring germinal or somatic DDR mutations was rucaparib. Its approval was based on the results of TRITON2, a single arm, phase 2 study investigating rucaparib in mCRPC patients with a DDR gene alteration who had received one prior taxane-based chemotherapy and one ARPI. The objective response rate (ORR) was 43.5%, including a complete response rate of 11.2% in BRCA1 and BRCA2 mutated patients [31], while there was no significant response in other DDR mutations carriers (ORR 13.5%) [64], leading to FDA accelerated approval of Rucaparib only for mCRPC patients with somatic or germline BRCA1 or BRCA2 mutation. The randomized phase 3 TRITON3 trial evaluated rucaparib compared to a physician’s choice of abiraterone, enzalutamide or docetaxel in mCRPC patients with BRCA1, BRCA2 or ATM mutations progressing after one prior ARPI. The primary endpoint was rPFS, which was first tested in BRCA-mutated population and then in intention to treat (ITT) population. The study met its primary endpoint by showing a benefit in rPFS in patients receiving rucaparib both in BRCA subgroup (HR 0.50, 95% CI 0.36–0.69, *p* < 0.001) and in ITT population (HR 0.61, 95% CI 0.47–0.80, *p* = 0.003). Importantly, in ATM patients, no benefit from rucaparib compared to physician’s choice was noted (8.1 vs. 8.1 months, HR 1.1, 95% CI 0.57–2.11). Interim OS was recently presented at ASCO GU 2023, where a trend in OS benefit in both subgroups was shown, although not meeting the statistical significance (BRCA subgroup: HR 0.81, 95% CI 0.58–1.12, *p* = 0.21; ITT population: HR 0.94, 95% CI 0.72–1.23, *p* = 0.67) [65] (Table 1). The most common adverse events related to rucaparib were fatigue (61%), nausea (50%), anemia (47%) and decreased appetite (36%). Most frequent grade ≥ 3 toxicity were anemia (24%), neutropenia (7%), fatigue (7%) and thrombocytopenia (6%). No case of myelodysplastic syndrome or acute myeloid leukemia have been reported. 

Olaparib is another PARPi that showed efficacy in PC. In the phase II single-arm TOPARP A trial, olaparib showed a high response rate (88%) in DDR mutated mCRPC patients [66]. The phase 2 randomized TOPARP B trial confirmed olaparib efficacy in mCRPC, especially in patients with BRCA1/2 aberrations [67]. In the randomized, open-label Phase 3 PROFOUND study, olaparib was evaluated in mCRPC harboring a genetic alteration in 15 prespecified HRR-related genes who had received a first-line ARPI. Patients were included in one of two cohorts depending on their qualifying gene alteration: BRCA1, BRCA2 or ATM-mutated patients were assigned to cohort A; patients with alterations in any of the other 12 genes were assigned to cohort B. In each cohort, patients were randomized in a 2:1 ratio to olaparib or the physician’s choice of enzalutamide or abiraterone (control arm). The results proved a longer PFS in the olaparib group compared to the control arm both in Cohort A (7.4 vs. 3.6 months, *p* < 0.001) and in the overall population (5.8 vs. 3.5 months, *p* < 0.001). These data led to the FDA approval of olaparib in patients with at least one alteration in the 15-gene panel (BRCA1, BRCA2, ATM, BRIP1, BARD1, CDK12, CHEK1, CHEK2, FANCL, PALB2, PPP2R2A, RAD51B, RAD51C, RAD51D, and RAD54L) [30]. Subsequent OS analysis showed that in cohort A, patients who received olaparib had a significantly longer duration of OS than those receiving control therapy (19.1 vs. 14.7 months, HR 0.69; 95% CI 0.50–0.97; *p* = 0.02), while in cohort B, OS was shorter in both arms (14.1 months with olaparib vs. 11.5 months with control therapy, HR 0.96; 95% CI 0.63–1.49) [68]. Based on this evidence, EMA approval of olaparib is limited to patients with BRCA1 and BRCA2 mutations.

Talazoparib is approved for germline BRCA-mutated breast cancer patients but remains an experimental treatment in PC. Talazoparib was evaluated in an mCRPC setting in the single-arm phase 2 TALAPRO-1 trial. This study enrolled mCRPC patients with a somatic or germinal mutation in one of eleven prespecified HRR-related genes and who had progressed on a prior taxane and at least one ARPI. Results showed an ORR of 29.8%, but with different rates depending on the HRR gene alteration group (46% in BRCA1-2 mutated, 25% in PALB2, 17% in ATM and no ORR observed in other patients). Median PFS was 5.6 months (11.2 months in BRCA1-2 mutated patients) [69]. Following these promising preliminary results, two randomized phase 3 studies testing talazoparib in patients with prostate cancer are ongoing: the TALAPRO-2 trial is evaluating talazoparib in combination with enzalutamide versus enzalutamide plus placebo as a first line treatment in mCRPC patients (results already presented at ASO 2023 for rPFS and FDA approval has been granted in August 2023), while the TALAPRO-3 trial is evaluating the same combination in mHSPC patients DDR gene-mutated.

Niraparib is a PARPi approved in ovarian cancer as a maintenance treatment but remains an investigational therapy in PC. GALAHAD was an open-label phase 2 trial of niraparib in mCRPC patients with a mutation in one of eight DDR gene panels (BRCA1, BRCA2, ATM, FANCA, PALB2, CHEK2, BRIP1, or HDAC2) that received at least one taxane-based treatment and one ARPI. Patients were allocated to cohorts on the basis of genetic aberrations (BRCA and non-BRCA mutations), and each cohort included patients with measurable and non-measurable disease. ORR in the measurable BRCA cohort was 34.2%. PFS and OS were longer in BRCA-mutated patients than in non BRCA (8.1 vs. 3.7 months and 13 vs. 9.6 months, respectively). Therefore, this trial established Niraparib anti-tumor activity in heavily pretreated mCRPC patients with DDR mutations, particularly in those with BRCA alterations [70].

## 5. Biological Rationale for the ARPI–PARP Inhibitor Combination

**Figure 2:**
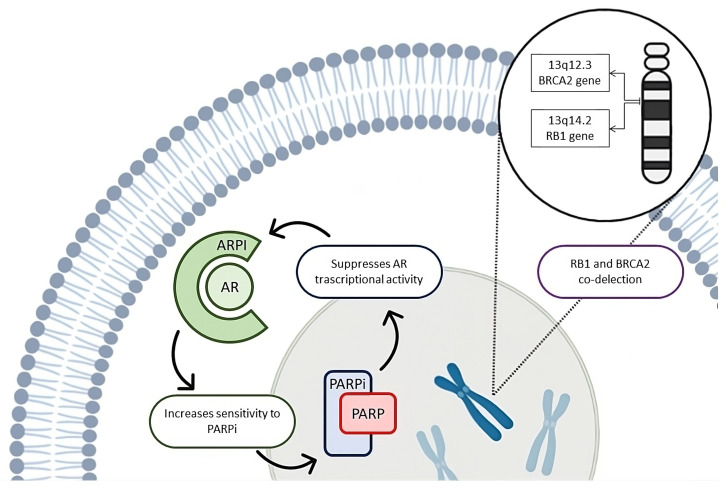
Three-fold rational of co-inhibiting AR and PARP regardless of DDR mutational status. Androgen receptor (AR) inhibition suppresses homologous recombination repair (HRR) genes expression, enhancing sensitivity to PARP inhibitors (PARPi). Simultaneously, the blockade of PARP may reduce AR transcriptional activity, hence increasing responses to androgen receptor pathway inhibitors (ARPI). In addition, some cases of metastatic prostate cancer show a more aggressive behavior and a weaker response to ARPI due to loss of RB transcriptional corepressor 1 (RB1) gene, which is often co-deleted with BRCA2, justifying the addition of a PARPi to treatment with ARPI in this subset of patients.

There is a strong biological rationale for the combination of a PARPi with an ARPI in patients with PC (Figure 2).

Firstly, AR signaling inhibition determined by the ARPI suppresses the expression of HRR genes, making cancer cells potentially susceptible to PARPi treatment. This phenomenon is called “BRCAness”, defined as the molecular feature that some sporadic cancers share with hereditary BRCA-mutation ones [71,72]. Analysis conducted on tissue samples from patients with resistant PC revealed an upregulation of HRR-related genes, including BRCA1, RAD54L and RMI2, which are implicated in the development of castration resistance. Preclinical studies showed that AR regulates a transcriptional program of DNA repair genes; therefore, its ARPI-induced downregulation compromises the ability of PC cells to repair DSBs and improves sensitivity to PARPi [32,71]. In addition, it is well known that ADT improves responses to radiotherapy in PC [73,74,75,76], and one of the possible mechanisms behind this evidence is that androgen-deprived cells receiving ionizing radiations are severely compromised in the ability to repair radiation-induced DSBs [77]. This means that castration promotes tumor cells radiosensitivity through down-regulating DSB repair systems, supporting the hypothesis of generating a synthetic lethality between ARPI and PARPi [78].

Secondly, PARP1 activity supports AR transcriptional function [79,80], and therefore, PARP inhibition is expected to reduce AR signaling activity and increase sensitivity to ARPI. A key mechanism of developing castration resistance and driving tumor progression is represented by molecular alterations of AR and in particular the generation of alternatively spliced variants of the full-length AR (AR-Vs), resulting in constitutive androgenic signaling because they lack the ligand-binding domain and the targeted drug site [81]. There is evidence showing that AR-Vs can regulate a DDR gene network critical for cell survival. Preclinical studies underline that PARP inactivation compromises the expression of AR-V-target genes, enhancing sensitivity to ARPI [79].

The third rationale supporting the benefit of combining PARPi with ARPI comes from the hypothesis that resistance to AR therapies may be related to a loss of RB transcriptional corepressor 1 (RB1) gene, which is often co-deleted with BRCA2. RB1 is located on chromosome 13q in close proximity to the BRCA2 gene. The co-deletion of RB1 and BRCA2 has been described in up to 50% of patients with metastatic disease in some series [82]. In PC cells, BRCA2-RB1 codeletion induces an epithelial-to-mesenchymal transition, which is associated with invasiveness and a more aggressive disease phenotype, which translates to shorter survival and shorter time on an ARPI [83]. A recent study has reported that BRCA2-RB1 codeletion is independently associated with the earlier development of metastases and shorter survival in patients with and without germline BRCA2 mutations [84]. The identification of this aggressive form offers potential for improved outcomes with earlier introduction of PARPi therapy [82].

These three biological rationales support a potential synergistic effect of the ARPI-PARPi combination, regardless of DDR mutational status, and represent an important opportunity for further investigations.

## 6. Clinical Implications of the Co-Inhibition of ARPI and PARP-Inhibitors

Several studies have investigated the efficacy of the ARPI and PARPi combination. A phase 2 trial of veliparib (a PARPi with weaker PARP-trapping activity compared to olaparib), in combination with abiraterone versus abiraterone alone found no significant difference in PSA response (72.4% vs. 63.9%; *p* = 0.27) or PFS (11 vs. 10.1 months; *p* = 0.99) in 148 mCRPC patients. The only exception was represented by patients with DDR mutations, who experienced significantly longer PFS compared to other subgroups in both treatment arms (mPFS 14.5 vs. 8.1 months; *p* = 0.025). This finding not only confirms the already-known benefit of PARPi in mutated HHR (mHRR) patients, but also demonstrates that HRR defects do not preclude benefit from ARPI [85].

The combination of olaparib and abiraterone was originally evaluated in a phase 2 randomized double-blind trial vs. abiraterone plus placebo. The trial enrolled 171 mCRPC patients who had previously received docetaxel, regardless of HRR alterations. Results showed that in the overall population median, rPFS was superior in patients treated with abiraterone plus olaparib compared with abiraterone plus placebo (median 13.8 vs. 8.2 months; HR 0.65, 95% CI 0.44–0.97, *p* = 0.034) [86]. Subgroup analysis by HRR status showed similar outcomes in rPFS in mHRR patients (n = 21, median PFS: 17.8 vs. 6.5 months in olaparib and placebo groups respectively, HR 0.74, 95% CI 0.26–2.12; *p* = 0.58) and in nonmutated HRR (nmHRR) patients (n = 35, median PFS: 15.0 vs. 9.7 months, HR 0.52, 95% CI 0.24–1.15; *p* = 0.11). These results suggested that synergy between PARP inhibition and AR pathway may exist independently of deleterious HRR alteration [87].

Based on these data, three phase 3 trials were initiated to investigate the role of ARPI-PARPi combination in first-line mCRPC: PROpel, MAGNITUDE, and TALAPRO-2. The three studies evaluated PARPi and ARPI combinations as first line of treatment for mCRPC and shared rPFS as a primary outcome, but otherwise had remarkably different designs, including the prospective or retrospective HRR testing, the stratifying factors, the percentage of patients with HRR and BRCA alterations or the prior use of ARPI. 

In the phase 3 PROpel trial, 796 mCRPC patients were randomized 1:1 to receive abiraterone with olaparib or placebo in first line setting. The primary endpoint was rPFS, while OS was a secondary endpoint. Patients were enrolled regardless of HRR mutation status. At the interim analysis [88], treatment with abiraterone plus olaparib significantly prolonged rPFS compared with abiraterone alone, irrespective of HRR status (24.8 vs. 16.6 months; HR 0.66, 95% CI 0.54–0.81; *p* value < 0.0001). Predefined subgroup analyses showed rPFS improvement across all subgroups, including candidates with HRR mutations (HR 0.50, 95% CI 0.34–0.73) and wild type (HR 0.76, 95% CI 0.60–0.97). These findings confirm the results of the previous phase 2 trial. Final OS data were recently published, showing a consistent trend toward OS benefit in ITT population with abiraterone plus olaparib compared to abiraterone plus placebo (median OS: 42.1 vs. 34.7 months, maturity 47.9%, HR 0.81, 95% CI 0.67–1.00, *p* = 0.0544) [89]. Patients with BRCA1/2 aberrations had significant superior survival with abiraterone plus olaparib compared to abiraterone plus placebo (HR 0.29, 95% CI 0.14–0.56), while nmHRR patients did not have a significant OS benefit with the combination (HR 0.89, 95% CI 0.7–1.14) [90]. The most common adverse events (AEs) in the abiraterone and olaparib arm were anemia, fatigue/asthenia, and nausea. Anemia was the most common AE, with an incidence of 49.7% in the olaparib group versus 17.7% in the control group. Grade 3 or higher anemia occurred in 60 patients (15.1%) in the abiraterone and olaparib arm and 13 patients (3.3%) in the abiraterone and placebo arm. Hypertension was the most common non-hematological toxicity with comparable incidence in both arms (3.8% vs. 3.5%). Some cases of venous thromboembolism were reported in both arms, with higher rates in abiraterone plus olaparib group compared to abiraterone alone (7.3% vs. 3.3%). As a result of this phase 3 trial, the EMA approved olaparib plus abiraterone/prednisone in mCRPC patients for whom chemotherapy is not clinically indicated, whereas the FDA gave approval only for BRCA-mutated mCRPC patients.

MAGNITUDE is a phase 3 trial in which 423 mCRPC patients were randomized to first-line treatment with abiraterone plus niraparib or placebo. The study population included two cohorts: the mHRR cohort, consisting of patients with pathogenic gene alterations in ≥1 of the following: ATM, BRCA1, BRCA2, BRIP1, CDK12, CHEK2, FANCA, HDAC2, or PALB2; and the nmHRR cohort, which included patients who had no detectable alterations in any of these genes. The primary endpoint of this study is rPFS. The first interim analysis (futility analysis) showed that the combination therapy significantly improves rPFS in the BRCA1/2 subgroup and in all mHRR patients, reducing the risk of progression or death by 47% (median PFS: 16.6 vs. 10.9 months; HR 0.53; 95% CI, 0.36 to 0.79; *p* = 0.001) and 27% (16.5 vs. 13.7 months; HR, 0.73; 95% CI, 0.56 to 0.96; *p* = 0.022) respectively, compared with abiraterone plus placebo. Niraparib with abiraterone delays time to initiation of cytotoxic chemotherapy (TTCC), time to symptomatic progression (TTSP), time to PSA progression (TTPP), and improves ORR in mHRR patients. Updated results from interim analysis 2 were recently presented at ASCO GU 2023 [91], confirming the meaningful benefit of niraparib plus abiraterone in BRCA1/2 population (rPFS 19.5 months vs. 10.9) and also in the mHRR cohort (HR 0.76; 95% CI, 0.60–0.97, *p* = 0.028). OS results are still immature.

In nmHRR patients, combination therapy showed no benefit in the composite end point of time to PSA progression and/or rPFS (HR, 1.09; 95% CI, 0.75–1.57), and futility was declared per the prespecified criteria [92].

The combination treatment was well tolerated, and the incidence of grade ≥ 3 AEs was 67.0% with niraparib + abiraterone and 46.4% with placebo + abiraterone. The most common grade 3 AEs were anemia (28.3% vs. 7.6%, with niraparib + abiraterone versus placebo + abiraterone, respectively) and hypertension (14.6% vs. 12.3%), followed by thrombocytopenia (6.6% vs. 2.4%) and neutropenia (6.6% vs. 1.4%).

The TALAPRO-2 study is a phase 3, double-blind, placebo-controlled trial aiming to evaluate talazoparib in combination with enzalutamide as a first-line treatment for patients with mCRPC with or without DNA damage repair alterations. This study has two co-primary endpoints: rPFS in all-comers (cohort 1) and in patients with DDR alterations (cohort 2). The first results obtained were recently published [93]. Combination treatment resulted in a 37% reduced risk of progression or death, with a clinically meaningful benefit regardless of HRR status (median rPFS: not yet reached vs. 21.9 months; HR 0.63, 95% CI: 0.51–0.78; *p* < 0.001). Longer PFS was reported in the combination arm compared to enzalutamide alone in both patients harboring alterations in HRR genes (median PFS: 27.9 vs. 16.4 months, HR 0.46 95% CI: 0.30–70, *p* < 0.001) and those with known absence of mutation in target genes (median PFS: not yet reached vs. 22.1, HR 0.66, 95% CI 0.49–0.91; *p* = 0.009). TTPP, TTCC and PFS2 are among the secondary endpoints being evaluated, and they all support the benefit of the combination therapy in the overall population. OS results are still immature [94]. Anemia was the most common AE of any grade, found in 66% of the talazoparib arm versus 17% in the control arm. Among the most common AEs resulting in a dose reduction of talazoparib, there were anemia (43.2%), neutropenia (15.1%) and thrombocytopenia (5.5%). A total of 8.3% of patients discontinued talazoparib due to anemia. Rare cases of myelodysplastic syndrome or acute myeloid leukemia were detected (Table 2). The combination of talazoparib and enzalutamide has been recently FDA approved based on the phase 3 data of TALAPRO-2 for HRR gene-mutated mCRPC.

To further evaluate the efficacy of the ARPI + PARPi combination versus ARPI or PARPi monotherapy in patients with germline or somatic HRR genes, the biomarker-selected, randomized, open-label, phase 2 BRCAAway trial is ongoing (NCT03012321). Patients with inactivating BRCA1, BRCA2 and/or ATM alterations were enrolled and randomized 1:1:1 to Arm 1 (abiraterone), Arm 2 (olaparib) or Arm 3 (olaparib + abiraterone). The preliminary results reported at ASCO 2022 showed that in mCRPC patients with inactivating BRCA1, BRCA2, and/or ATM alterations, abiraterone + olaparib resulted in longer PFS (12-month PFS rate: 40% (95% CI 0.21–0.77), 49% (0.29–0.82), 95% (0.86–1.0), in Arms 1, 2 and 3, respectively) and better PSA response (≥50% PSA decline: 79% in Arm 1, 81% in Arm 2, and 90% in Arm 3) versus either agent alone [95].

A recent systematic review and metanalysis, aiming to synthesize evidence from randomized trials assessing the efficacy and safety of PARPi + ARPI combinations for the first-line treatment of mCRPC was published. In this metanalysis, data from TALAPRO 2 and PROpel showed a clear rPFS benefit of PARPi + ARPI for first-line treatment of mCRPC, independent of HRR status (pooled HR 0.62, 95% CI 0.53–0.72). Subgroup analysis confirmed rPFS benefit both in mHRR mCRPC patients (HR 0.57, 95% CI 0.42–0.78) and in nmHRR (HR 0.76, 95% CI 0.65–0.90), although the greatest benefit was observed in BRCA-mutated (mBRCA) mCRPC patients (HR 0.36, 95% CI 0.16–0.82). The pooled HR for OS was 0.84 (95% CI 0.72–0.98), indicating a 16% reduction in the risk of death among patients who received the combination [96], although these data do not take into account the ASCO updates mentioned above.

## 7. Future Perspectives and Open Questions

Several ongoing clinical trials are evaluating the combination of ARPI and PARPi in patients with PC (Table 3). The CASPAR trial (NCT04455750) is a randomized phase 3 study in which patients with mCRPC independent of HRR mutational status are randomized to enzalutamide plus rucaparib or enzalutamide plus placebo. It is the only combination study in which OS and rPFS are both co-primary endpoints, aiming to answer the question of whether concomitant therapy is more effective than sequential therapy with PARPi. 

Other ongoing studies are evaluating the PARPi and ARPI combination in earlier PC settings. The AMPLITUDE trial (NCT04497844) is evaluating niraparib in combination with abiraterone versus abiraterone alone in patients with germline or somatic HRR alterations in the mHSPC setting. TALAPRO-3 (NCT04821622) is a study of talazoparib with enzalutamide versus enzalutamide alone in men with deleterious DDR gene-mutated mHSPC. Both these trials have rPFS as a primary outcome and will provide further data about the benefit of using such a combination approach in earlier stages in mutated patients. Meanwhile, talazoparib in combination with ARPI in mHSPC is being tested in two phase 2 trials (NCT04734730, NCT04332744), where eligible patients are unselected for DDR alterations. These studies will provide data about a combination approach in non-mutated mHSPC and will help identify subgroups of patients who can benefit from treatment intensification.

In the mHSPC setting, olaparib, along with abiraterone, is being studied in the mHRR population in a phase 2 trial (NCT05167175). Additional studies are being put forward, including trials with different PARPi: for instance, within the mCRPC scenario, ongoing research with fuzuloparib (NCT04691804) and TQB3823 (NCT05405439) is conducted in unmutated populations.

Clinical studies go beyond the combination of PARPi and ARPI and the role of HRR status. Ongoing trials are also examining the potential synergistic lethality that could arise from combining PARPi with other agents. These combination strategies aim to expand the population of patients who can benefit from PARPi therapy and to overcome or delay mechanisms of acquired resistance to PARPi.

In particular PARPi has been investigated in combination with immunotherapy. Preclinical data show that unrepaired DNA damages induced by PARPi may alter the tumor immune microenvironment through various molecular and cellular mechanisms. These include increased genomic instability, activation of immune pathways, and increased PD-L1 expression on cancer cells [97]. These alterations could potentially enhance responsiveness to immune checkpoint inhibitors (ICIs). Multiple trials are exploring the combination of PARPi with ICIs in mCRPC. These include anti-PD1 agents like pembrolizumab (KEYLYNK-010 NCT03834519), nivolumab (CheckMate9KD NCT03338790), and cetrelimab (NCT03431350), as well as anti-PDL1 agents such as avelumab (JAVELIN PARP NCT03330405) and durvalumab (NCT03810105).

## 8. Discussion

The AR represents a crucial target in PC, and therefore, various ARPIs (e.g., abiraterone, enzalutamide, apalutamide and darolutamide) demonstrated efficacy and were approved in different disease settings. However, there is a strong inter- and intra-patient heterogeneity in PC that may undermine the efficacy of such therapies. For example, it is well known that not all PC are AR-dependent, showing an early resistance to treatment with ARPI [98,99]. Knowing these limitations is crucial in order to encourage the development of new therapeutic strategies. PARPi are a new valid therapeutic option in mCRPC patients with DDR mutations [30]. However, only about 20% of mCRPCs harbor DNA repair defects and are eligible for PARPi monotherapy. In clinical practice, the percentage of patients who benefit from this treatment could be even lower, due to pre-analytical, analytical and post-analytical difficulties in the detection of DDR mutations. In fact, the optimal tissue for genetic testing may depend on sample availability, tumor archiving and storage period. In addition, interpretation and classification of variants in the DRR pathway are challenging. Additional limitations in some countries could be costs and availability of genetic testing [36,100].

Preclinical data suggest a strong biological rationale supporting the ARPI + PARPi combination use, regardless of DDR mutational status in patients with PC. Combining two drugs that target different pathways may help overcome single mechanisms of resistance [101]. This is particularly important when talking about AR resistance, because in such cases, adding PARPi may reduce AR transcriptional activity and increase responses to ARPI [79,80]. However, the clinical evidence is much less convincing. Clinical trial results showed in the mCRPC setting that ARPI and PARPi combination improved rPFS compared to ARPI monotherapy in patients with HRR mutation, while evidence in non-mutated patients is contradictory.

The MAGNITUDE trial showed no rPFS benefit in the nmHRR population, and the nmHRR cohort was closed early for futility. In contrast, PROpel and TALAPRO-2 demonstrated a benefit in rPFS also in patients without detectable HRR genetic aberrations, although more modest than those with DDR mutations, as confirmed by Messina’s metanalysis [96].

To better understand these diverging results, it is necessary to point out the critical differences between these two trials. First, MAGNITUDE was a biomarker-stratified study where HRR mutational status was prospectively determined before randomization, while PROpel enrolled biomarker-unselected patients, and analysis by HRR status was exploratory. In addition, the method of defining HRR mutational status and the gene panel used to classify HRR alterations in each study was not identical. Furthermore, the two studies used different PARPi in combination with abiraterone (niraparib in MAGNITUDE, olaparib in PROpel), and potential synergies cannot be assumed to be identical. Also, while the olaparib dose in PROpel was the same as that from monotherapy studies (300 mg twice daily), the dose of niraparib in MAGNITUDE was lower (200 mg once daily compared with 300 mg once daily in monotherapy studies); hence, the potency of PARP inhibition by niraparib could be compromised. 

Additionally, the benefit of the combination therapy observed in PROpel in rPFS is not confirmed by OS data. Recent results demonstrated a trend towards OS benefit in the ITT population, but statistical significance was not met. Furthermore, the subgroup analyses showed weak results in both mHRR non BRCA1/2 patients and in nmHRR patients. The prolonged survival in the ITT population may be the effect of the strong benefit observed in BRCA1/2 mutated patients, which are the patients who have the most advantage from this combination therapy.

To date, PARPi are used in patients with mCRPC previously treated with an ARPI in accordance with the pivotal trials. The results of these combination studies may anticipate the use of PARPi as first-line therapy in the mCRPC setting in combination with ARPI. However, OS data are needed to confirm the efficacy of this combination in this setting. In addition, the final results of these and other ongoing studies will help us to identify the patients most likely to benefit from this combination therapy. As for now, the results demonstrate a clear benefit of the ARPI and PARPi combination in patients with HRR gene alterations, especially in BRCA1/2 aberrations, and therefore, in these patients, this combination should represent the standard of care as first-line therapy in the mCRPC setting. 

Furthermore, as the Capture has shown, if BRCA patients have significantly worse outcomes than patients with mutations in HRR non-BRCA, and if those with alterations in HRR non-BRCA genes have worse outcomes than non-mutated patients, early screening for HRR mutations, particularly BRCA1/2, is crucial to anticipate a target treatment of mCRPC and improve prognosis [52]. Therefore, it should be mandatory, as recommended by international guidelines, to perform a molecular analysis in all patients with metastatic PC [102].

In line with expectations, the combination of ARPI and PARPi in clinical trials had higher AEs rates, dose interruptions and dose reductions. The most common G3-4 AEs in combination treatment were hematological toxicities, including anemia, thrombocytopenia and neutropenia, which are known to be attributed to PARPi. Hypertension was a common G3-4 non-hematological AE, with similar incidence rates in experimental and control arms, being that it is a well-known side effect of ARPI [15,16,17,18]. Other non-hematological AE included asthenia and nausea, which were reported to be more frequent in patients treated with PARPi [96,103]. Although in PROpel the abiraterone plus olaparib arm showed a higher rate of venous thromboembolic events compared to abiraterone alone, the overall rate of cardiovascular events was comparable between treatment arms and consistent with the incidence reported in PC patients in the literature [69,104]. In conclusion, toxicities associated with combination treatment are not particularly different from those emerging from PARPi monotherapies [105], although their incidence is higher. This means that the AE profile remains consistent with the known individual toxicity of each drug, and their combination does not amplify the toxicity of either drug.

An important conclusion from PROPEL, TALAPRO-2 and MAGNITUDE is that a benefit hierarchy aligned with known biology can be established: BRCA2-deficient tumors are those who benefit the most from combination therapy, followed by all HRR-deficient tumors, unselected patients and eventually HRR-proficient tumors [106].

Finally, these considerations are valid only in patients receiving ADT monotherapy in mHSPC and nmCRPC since nearly all patients in the clinical trials had not received a prior ARPI. This makes it difficult to reproduce the results of these studies in current clinical practice since ADT monotherapy is no longer the standard of care in mHSPC and nmCRPC with a PSA doubling time < 10 months, and therefore, almost all patients fit for an ARPI will receive it at an earlier stage. In these patients who have already received a prior ARPI, the use of sequential PARPi therapy should continue to be the standard of care. In general, to date, we do not know whether ARPI + PARPi combination therapy or ARPI–PARPi sequential therapy is to be preferred in patients with mCRPC, and the ongoing BRCAAway study that is comparing these two different therapeutic strategies will help us in this unmet clinical need.

## 9. Conclusions

There is strong biological evidence that the co-inhibition of AR and PARP may result in therapeutic synergy. To date, it seems that the patients who benefit most from the ARPI + PARPi combination are those with HRR mutations and, in particular, patients with BRCA 1/2 mutations; however, we must wait for the final results of the ongoing studies to confirm these initial findings and to understand how to insert this new therapeutic approach into the therapeutic landscape of PC.

## Figures and Tables

**Table 1:** Clinical trials with PARP inhibitors in monotherapy in mCRPC.

Study	Phase	Patients	Treatment	Response Rate	PFS	OS
TOPARP A (NCT01682772)	2	mCRPC with prior chemotherapy unselected for DDR gene aberrations (n = 50), including biomarker negative (n = 33) and biomarker positive (n = 16)	Single arm: olaparib 400 mg BID	33% in all evaluable patients 88% in biomarker positive vs. 6% in biomarker negative patients.	9.8 months in biomarker positive vs. 2.7 months in biomarker negative (HR 0.24; 95% CI 0.11–0.50; *p* < 0.001)	13.8 months in biomarker positive vs. 7.5 months in biomarker negative (HR 0.47; 95% CI 0.22–1.021; *p* = 0.05)
TOPARP B (NCT01682772)	2	mCRPC with prior chemotherapy selected for DDR gene aberrations (n = 98)	Olaparib 400 mg BID (n = 49) vs. Olaparib 300 mg BID (n = 49)	400 mg BID cohort: 54.3% 300 mg BID cohort: 39.1%	400 mg BID cohort: 5.5 months (95% CI 4.4–8.3) 300 mg BID cohort: 5.6 months (95% CI 3.7–7.7)	400 mg BID cohort: 14.3 months (95% CI 9.7–18.9) 300 mg BID cohort: 10.1 months (95% CI 9.0–17.7)
PROfound (NCT02987543)	3	mCRPC with prior NHA, chemotherapy-naïve, selected for DDR gene aberrations (n = 387) Cohort A (n = 245): at least one alteration in BRCA1, BRCA2, or ATM Cohort B (n = 142): alterations in any of the other DDR genes	Experimental arm: olaparib 300 mg BID Control arm: physician choice ARPI (enza 160 mg/day or AA 1000 mg/day) Cohort A: Olaparib (n = 162) vs. physician choice ARPI (n = 83) Cohort A+B: Olaparib (n = 256) vs. physician choice ARPI (131)	Cohort A: 33% in experimental arm vs. 2% in control arm Cohort A+B: 22% in experimental arm vs. 4% in control arm	Cohort A: 7.4 vs. 3.6 months (HR 0.34; 95% CI 0.25–0.47; *p* < 0.001) Cohort A+B: 5.8 vs. 3.5 months (HR 0.49; 95% CI 0.38–0.63; *p* < 0.001)	Cohort A: 19.1 vs. 14.7 months (HR 0.69; 95% CI 0.50–0.97; *p* = 0.02) Cohort A+B: 17.3 vs. 14.0, (HR 0.55; 95% CI 0.29–1.06, crossover adjusted analysis)
TRITON2 (NCT02952534)	2	mCRPC with prior NHA and chemotherapy, selected for DDR gene alterations (n = 203) BRCA1/2 subgroup: at least one germinal or somatic aberration in BRCA1 or BRCA2 (n = 115)	Single arm: rucaparib 600 mg BID	43.5% in BRCA1/2 subgroup 13.5% in other DDR gene alterations	9.0 months (95% CI 8.3–13.5 months) in BRCA1/2 subgroup	12-month OS 73% (95% CI 62.9%–80.7%, maturity 41%) in BRCA1/2 subgroup
TRITON3 * (NCT02975934)	3	mCRPC with prior NHA, chemotherapy-naïve, selected for mutations in any of BRCA1/2 or ATM genes (n = 405) BRCA1/2 subgroup: at least one germinal or somatic aberration in BRCA1 or BRCA2 (n = 302)	Experimental arm: rucaparib 600 mg BID (n = 270) Control arm: physician choice of ARPI (n = 60) or Docetaxel (75)	-	ITT population: 10.2 vs. 6.4 months (HR 0.61, 95% CI 0.47–0.80, *p* = 0.0003) BRCA1/2 subgroup: 11.2 vs. 6.4 months (HR 0.50, 95% CI 0.36–0.69; *p* < 0.0001)	ITT population: 23.6 months vs. 20.9 months (HR 0.94, 95% CI 0.72–1.23, *p* = 0.67, maturity 59%) BRCA1/2 subgroup: 24.3 months vs. 20.8 months (HR 0.81, 95% CI 0.58–1.12; *p* = 0.21, maturity 54%)
TALAPRO 1 (NCT03148795)	2	mCRPC with prior NHA and chemotherapy, selected for DDR gene aberrations (n = 128) BRCA1/2 subgroup: at least one germinal or somatic aberration in BRCA1 or BRCA2 (n = 61)	Single arm: talazoparib 1 mg/day	29.8% in all evaluable patients 45.9% in BRCA1/2 subgroup	5.6 months (95% CI 3.7–8.8) BRCA1/2 subgroup: 11.2 months (95% CI 7.5–19.2)	16.4 months (95% CI 12.2–19.9)
GALAHAD (NCT02854436)	2	mCRPC with prior NHA and chemotherapy, selected for DDR gene aberrations (n = 223) BRCA cohort: germline pathogenic or biallelic pathogenic alterations in BRCA1 or BRCA2 (n = 142), including measurable (n = 76) and non-measurable disease (n = 66) Non-BRCA cohort: alterations in any of the other DDR genes (n = 81), including measurable (n = 47) and non-measurable disease (n = 34)	Single arm: niraparib 300 mg/day	34.2% in measurable BRCA cohort 10.6% in measurable non-BRCA cohort	BRCA cohort: 8.08 months (95% CI 5.55–8.38) Measurable BRCA cohort: 5.52 months (95% CI 5.29–7.59) Non-BRCA cohort: 3.71 months (95% CI 1.97–5.49)	BRCA cohort: 13.01 months (95% CI 11.04–14.29) Measurable BRCA cohort: 10.87 months (95% CI 9.49–13.77) Non-BRCA cohort: 9.63 months (95% CI 8.05–13.44)

AA: abiraterone acetate; ARPI: androgen receptor pathway inhibitor; BID: bis in die; CI: confidence interval; DDR: DNA damage repair; Enza: enzalutamide; HR: hazard ratio; ITT: intention to treat; NHA: new hormonal agents; PFS: progression free survival; OS: overall survival. * TRITON 3 results are immature.

**Table 2:** Clinical trials with PARPi and ARPI in mCRPC.

Study	Phase	Patients	Treatment	Response Rate	PFS	OS
PROpel (NCT03732820)	3	mCRPC. Chemotherapy and ARPI allowed in mHSPC setting. No AA. Other NHAs allowed if stopped >12 months prior to enrollment. Patients unselected for DDR mutation (n = 796) AA + olaparib (n = 399) vs. AA + pbo (n = 397)	1:1 randomisation. Experimental arm (AA + olaparib): AA 1000 mg/day + prednisone 5 mg BID + olaparib 300 mg BID. Control arm (AA + pbo): AA 1000 mg/day + prednisone 5 mg BID + pbo.	40.3% AA + olaparib: ORR 58.4% (94/161) AA + pbo: ORR 48.1% (77/160) (OR 1.60; 95% CI, 1.02–2.53)	rPFS AA + olaparib vs. AA + pbo. ITT population: 24.8 vs. 16.6 months HR 0.66 (95% CI 0.54–0.81) *p* < 0.0001 mHRR: NR vs. 13.9 months, HR 0.5 (95% CI 0.34–0.73) nmHRR: 24.1 vs. 19 months, HR 0.76 (95% CI 0.6–0.97) mBRCA: NR vs. 8.4 months, HR 0.23 (95% CI 0.12–0.43)	47.9% maturity OS 42.1 vs. 34.7 months, HR 0.81 (95% CI 0.67–1); *p* = 0.0544 mHRR: NR vs. 28.5 months, HR 0.66 (95% CI 0.45–0.95) nmHRR: 42.1 vs. 38.9 months, HR 0.89 (95% CI 0.7–1.14) mBRCA: NR vs. 23 months, HR 0.29 (95% CI 0.14–0.56)
MAGNITUDE (NCT03748641)	3	mCRPC. Docetaxel and AA allowed in mHSPC. AA in m0CRPC allowed if given <4 months. Patients unselected for DDR mutation (n = 946) Cohort 1 (mHRR cohort, n = 423): niraparib + AA (n= 212) vs. AA + pbo (n = 211) Cohort 2 (nmHRR cohort, n = 247): niraparib + AA (n= 117) vs. AA + pbo (n = 116), closed for futility	1:1 randomisation. Experimental arm (niraparib + AA): niraparib 200 mg/daily + AA 1000 mg/day + prednisone 5 mg BID Control arm (pbo + AA): AA 1000 mg/day plus prednisone 5 mg BID + pbo	mHRR: niraparib + AA ORR 59.7% (55/92) vs. pbo + AA ORR 28% (28/82). RR 2.13, *p* < 0.001 BRCA1/2: niraparib + AA ORR 51.8% (29/56) vs. pbo + AA ORR 31.3% (15/48). RR 1.66, *p* = 0.04	mHRRm: 16.7 vs. 13.7 months HR 0.76 (95% CI 0.60–0.97); *p*= 0.0280 mBRCA: 19.5 vs. 10.9 months HR 0.55 (95% CI 0.39–0.78); *p* = 0.0007 nmHRRm HR 1.09; (95% CI 0.75 to 1.57); *p* = 5.66	mHRR: HR 1.01 (95% CI 0.75–1.36); *p* = 0.948 mBRCA: 0.88 (0.58, 1.34) *p* = 0.5505
TALAPRO-2 (NCT03395197)	3	mCRPC. Docetaxel and AA allowed in mHSPC. Patients selected for DDR mutation (n = 805) Talazoparib + enza (n = 402) vs. pbo + enza (n = 403)	1:1 randomisation. Experimental arm (talalazoparib + enza): talazoparib 0.5 mg/daily + enza 160 mg/daily Control arm (pbo + enza): enza 160 mg/daily + pbo	Talazoparib + enza: ORR 62% (74/120), (95% CI 52.4–70.4) vs. pbo + enza: ORR 44% (58/132), (95% CI 35.3–52.8); *p* = 0.005	ITT population: NR vs. 21.9 months HR 0.66 (95% CI 0.49–0.91); *p* = 0.009 mHRR: 27.9 vs. 16.4 months, HR 0.46 (95% CI 0.3–0.7); *p* < 0.01 nmHRR: NR vs. 22.5 months, HR 0.70 (95% CI 0.54–0.89): *p* = 0.04 mBRCA: HR 0.23 (95% CI 0.10–0.53); *p* < 0.001	36.4 vs. NR, HR 0.89 (95% CI 0.69–1.14) *p* = 0.35 (31% maturity)

AA: abiraterone acetate; ARPI: androgen receptor pathway inhibitor; BID: bis in die; CI: confidence interval; DDR: DNA damage repair; Enza: enzalutamide; HR: hazard ratio; HRR homologous recombination repair; ITT: intention to treat; mBRCA: mutated BRCA; mHRR: mutated homologous recombination repair; mHSPC: metastatic hormone-sensitive prostate cancer; NHA: new hormonal agents; nmHRR: non-mutated homologous recombination repair; NR: not reached; Pbo: placebo; PFS: progression free survival; OS: overall survival; RR: relative risk.

**Table 3:** Ongoing trials testing PARPis and ARPI combination both in mHSPC and mCRPC.

Study	Phase	Setting	Treatment Arms	HHR Mutational Status	Primary Endpoint
CASPAR trial (NCT04455750)	3	mCRPC with no prior chemotherapy or NHA in castration resistant setting (Docetaxel or NHA treatment allowed in mHSPC)	Experimental arm: rucaparib 600 mg BID + enza 160 mg/die Control arm: pbo + enza 160 mg/die	Unselected	rPFS and OS
AMPLITUDE trial (NCT04497844)	3	First line mHSPC	Experimental arm: niraparib 200 mg/die + AA 1000 mg/die + prednisone 5 mg BID Control arm: abiraterone 1000 mg/die + prednisone 5 mg BID + pbo	Selected	rPFS
TALAPRO-3 trial (NCT04821622)	3	First line mHSPC	Experimental arm: talazoparib 0.5 mg/die + enza 160 mg/die Control arm: pbo + enza 160 mg/die	Selected	rPFS
NCT04734730	2	First line mHSPC	Single arm: talazoparib 1 mg/die + AA 1000 mg/die + prednisone 5 mg BID	Unselected	PSA nadir < 0.2
ZZ First Trial (NCT04332744)	2	First line mHSPC	Experimental arm: talazoparib 0.5 mg/die + enza 160 mg/die Control arm: pbo+ enza 160 mg/die	Unselected	PSA-CR
NCT05167175	2	First line mHSPC	Single arm: olaparib 300 mg BID + AA 1000 mg/die + prednisone 5 mg BID	Selected	rPFS
NCT04691804	3	mCRPC with any systemic anti-tumor treatment during the mCRPC stage or non-metastatic CRPC stage	Experimental arm: fuzuloparib 150 mg BID + AA 1000 mg/die+ prednisone 5 mg BID Control arm: pbo + AA 1000 mg/die + prednisone 5 mg BID	Cohort 1: unselected Cohort 2: selected	rPFS
NCT05405439	1b/2	mCRPC with no prior NHA in mHSPC and nmCRPC.	Experimental arm: TQB3823 tablets (PARPi) + AA 1000 mg/die + prednisone 5 mg BID	Unselected	DLT, RP2D, rPFS
PETRANHA (NCT05367440)	1/2	mPC with no prior platinum or NHA	Arm 1: AZD5305 tablets (PARPi) + enza 160 mg/die Arm 2: AZD5305 tablets (PARPi) + AA 1000 mg/die + prednisone 5 mg BID Arm 3: AZD5305 tablets (PARPi) + daro 600 mg BID	Unselected	DLT, ORR, DoR, rPFS
NCT04108247	1	mCRPC with 4 weeks of wash out of any anti-tumor therapy	Experimental arm: SHR3162 (PARPi) + AA 1000 mg/die + prednisone 5 mg BID	Unselected	Incidence of AE and PK characteristics

AA: abiraterone acetate; AE adverse events; BID: bis in die; Daro: darolutamide DLT: dose-limiting toxicity; DoR: duration of response; Enza: enzalutamide; HRR: homologous recombination repair; MTD: maximum tolerated dose; NHA: new hormonal agent; ORR Objective response rate; PK: pharmacokinetic; PSA-CR: prostate specific antigen complete response (defined as the percentage of patients with PSA < 0.2 ng/mL divided by the number of patients in the analysis set); Pbo: placebo; rPFS: radiographic progression free survival; OS: overall survival; RP2D: recommended phase II dose.
